# LiFePO_4_-Graphene Composites as High-Performance Cathodes for Lithium-Ion Batteries: The Impact of Size and Morphology of Graphene

**DOI:** 10.3390/ma12060842

**Published:** 2019-03-13

**Authors:** Yanqing Fu, Qiliang Wei, Gaixia Zhang, Yu Zhong, Nima Moghimian, Xin Tong, Shuhui Sun

**Affiliations:** 1 Institut National de la Recherche Scientifique-Énergie Matériaux et Télécommunications, Varennes, QC J3X 1S2, Canada; yanqing.fu@emt.inrs.ca (Y.F.); qiliang.wei@emt.inrs.ca (Q.W.); xin.tong@emt.inrs.ca (X.T.); 2 NanoXplore Inc., 25 Montpellier Blvd., Saint-Laurent, QC H4N 2G3, Canada; yuzhong424@gmail.com (Y.Z.); moghimian@nanoxplore.ca (N.M.)

**Keywords:** LiFePO_4_ cathode material, graphene additive, lithium-ion batteries, commercial materials, promising performance

## Abstract

In this work, we investigated three types of graphene (i.e., home-made G, G V4, and G V20) with different size and morphology, as additives to a lithium iron phosphate (LFP) cathode for the lithium-ion battery. Both the LFP and the two types of graphene (G V4 and G V20) were sourced from industrial, large-volume manufacturers, enabling cathode production at low cost. The use of wrinkled and/or large pieces of a graphene matrix shows promising electrochemical performance when used as an additive to the LFP, which indicates that the features of large and curved graphene pieces enable construction of a more effective conducting network to realize the full potential of the active materials. Specifically, compared to pristine LFP, the LFP/G, LFP/G V20, and LFP/G V4 show up to a 9.2%, 6.9%, and 4.6% increase, respectively, in a capacity at 1 C. Furthermore, the LFP combined with graphene exhibits a better rate performance than tested with two different charge/discharge modes. Moreover, from the economic and electrochemical performance view point, we also demonstrated that 1% of graphene content is optimized no matter the capacity calculated, based on the LFP/graphene composite or pure LFP.

## 1. Introduction

Driven by serious global environmental issues, coupled with increasing fossil fuel costs, research and investment in the development of sustainable energies, such as wind, solar, hydroelectricity, biology, and geothermal energies, have been emerging among the global trends. Due to the intermittent and regional characteristics, these sustainable energies, need to be efficiently stored for various practical applications like backup power tools, electronic devices, and hybrid electric vehicles (HEVs). Energy-storage technology with good properties has been developed quite rapidly over the last decade. Currently, the lithium-ion battery (LIB) is one of the most promising energy storage systems because of its outstanding electrochemical performance and high capacity [1,2]. Furthermore, the development of LIBs is proceeding very quickly in response to the sharp demand from EVs and HEVs [3]. To meet such a strong demand in higher-capacity storage and power performance, the rate capability and energy density of LIB must be improved, which urges on the discovery of the innovative cathode and anode [4]. Since being reported by Padhi et al. [5], olivine lithium iron phosphate (LiFePO_4_ or LFP) has attracted significant attention as the most promising cathode candidate for LIBs, mainly due to its significant advantages, such as ease of synthesis, low cost, high theoretical capacity (170 mA h g^−1^), flat voltage plateau (ca. 3.4 V versus Li^+^/Li), environmental benignity, and high safety [6,7,8]. Nevertheless, the poor electronic conductivity of LFP (about 10^−9^ S cm^−1^ at 30 °C) limits their applications, especially on high-powered battery systems. Current strategies to enhance the electrochemical performance of LFP include carbon addition in LFP [9,10,11,12,13], metal doping [14,15,16,17], and LFP particle size reduction [18,19,20]. Carbon addition has been extensively used in the industry because the conductive carbon increases the electron migration rate during the charge/discharge processes [21]. Conventional carbon additives, such as carbon black and carbon coatings, possess relatively low electronic conductivity when compared with more crystalline forms of carbon [22]. Recently, graphene has taken the spotlight in LFP research because it possesses several desirable features, including high surface area and excellent electronic conductivity, for improving the electrochemical performance of LIBs. Several reports have demonstrated some enhancement in rate capacity using the LFP material combined with the graphene as the cathode. The significant capacity increase is attributed to the contribution of the graphene sheets to enhance the electron migration rate in LFP cathodes [23,24,25,26,27,28].

Therefore, the addition of graphene in an LFP composite significantly improves the electrochemical performance of LIBs. However, the impact of the graphene size and morphology on the electrochemical performance of LFP has been rarely reported. Herein, we reported the detailed investigation of the effects of size and morphology of graphene on electrochemical performance of LFP. In this research, the graphene materials as additives were added into LFP powder to form a cathode by simply physical mixing. The results also revealed the optimal ratio of graphene in the cathode and improved performance of various charge/discharge modes. In addition, most of the results came from the graphene materials synthesized at the research lab, which are not suitable for mass production, and the cost of the product is extremely high, hindering the commercialization of graphene materials. Herein, the LFP powder used in this work was from YANTAI ZHUONENG-BATTERY MATERIAL CO., LTD., while two types of commercial graphene materials (i.e., G V4 and G V20) from NanoXplore Inc. were investigated and compared with home-made graphene. Importantly, these three types of graphene present different sizes and morphology, and therefore, they provide a platform to investigate the effects of the size and morphology of graphene on the electrochemical performance of LFP. The results show that the features of large and curved graphene pieces enable construct a more effective conducting network to realize the full potential of the active materials. Moreover, from an economic and electrochemical performance view point, the optimized graphene content is proposed.

## 2. Experimental Section

### 2.1. Synthesis of LFP/Graphene Composites

**Figure 1:**
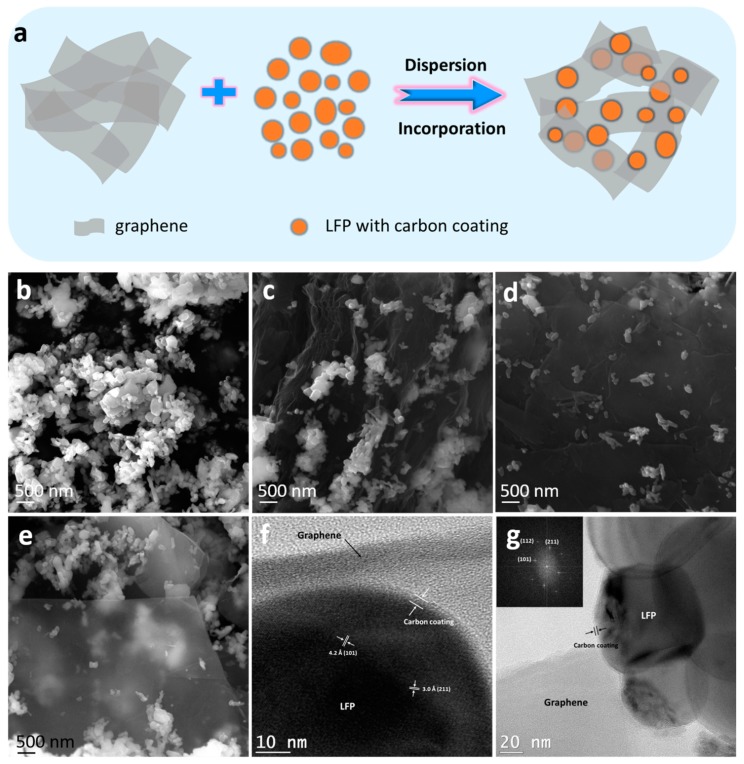
(**a**) Schematic of the synthetic process for a lithium iron phosphate (LFP)/graphene composite. SEM images of (**b**) pristine LFP, (**c**) LFP/G, (**d**) LFP/G V20, and (**e**) LFP/G V4 samples. TEM images (**f**,**g**) of LFP/graphene; the inset in (**g**) is the SAED pattern of the sample.

In this work, three types of graphene were involved in the preparation of LFP/graphene composites. Two grades of low-cost graphene [29], namely, heXo-G V4 (G V4) and heXo-G V20 (G V20), were from an industrial manufacturer (NanoXplore, Montreal, Canada) (NanoXplore Inc. https://www.nanoxplore.ca). The third type of graphene consisted of home-made graphene nanosheets (G), which were prepared with graphite oxidation, thermal exfoliation, and chemical reduction with natural flake graphite as the starting material [30]. In addition, the LFP (with 1.46% carbon coating) used in this work was from Yantai Battery Material Co., Ltd (Shandong, China). Using the commercial LFP and graphene could lower the cost of the LFP/graphene samples, which is favourable for commercialization. LFP/graphene samples were prepared using a simple method. Take the LFP/G-1% sample as an example. Typically, 0.005 g of graphene was dispersed into 60 mL absolute ethanol by sonication, and then 0.5 g of LFP nanoparticles was added into it by further sonication. Finally, the precipitate of LFP/graphene was collected after drying. The corresponding illustration of the synthetic process is shown in Figure 1a. The LFP/G-0%, LFP/G-2%, and LFP/G-4% composites were obtained in the same way.

### 2.2. Physical Characterizations

The crystal structure of the prepared samples was characterized by X-ray diffraction (XRD, Bruker D8 Advanced Diffractometer, Cu Kα radiation) (Bruker, Billerica, USA). The morphological structures were observed by scanning electron microscopy (SEM, Lyra3 SEM-FIB by Tescan) (Tescan, Brno, Czech Republic) and transmission electron microscopy (TEM, JEOL 2100F TEM) (JEOL Ltd., Tokyo, Japan). Energy dispersive X-ray spectroscopy (EDX) (Tescan, Brno, Czech Republic) is a chemical microanalysis technique used in conjunction with SEM.

The X-ray absorption near edge structure (XANES) measurements were performed at the Canadian Light Source (CLS), located at the University of Saskatchewan. The Li K-edge and P L-edge XANES were measured on the Variable Line Spacing Plane Grating Monochromator (VLS-PGM) (Canadian Light Source, Saskatoon, Canada) beamline. The XANES spectra were collected in both total electron yield (TEY) and fluorescent yield (FLY) mode (only FLY is presented in this work) with a chamber pressure above 1 × 10^−7^ torr at room temperature. The beamline slits’ sizes were 50 μm × 50 μm with an instrumental resolution of E/ΔE > 10,000. The data were normalized by the I_0_ current, which was simultaneously measured with the FLY, by monitoring the drain current emitted from a Nickel mesh (90% transmission) placed in front of the sample.

The Fe K-edge XANES was obtained at the soft X-ray micro characterization beamline (SXRMB). It can provide 10^11^ photons/s at 100 mA with a resolution higher than 10,000 (*E*/Δ*E*). The spectra were normalized with respect to the edge height after subtracting the pre-edge and post-edge backgrounds using Athena software.

### 2.3. Electrochemical Measurements

The button-type cells were assembled in an argon-filled glove box (VacuumTechnologyInc., Massachusetts, USA), where water and oxygen concentrations were kept at less than 5 ppm. The working electrodes were fabricated by mixing 80 wt % of active materials, 5 wt % of acetylene black, 5 wt % of graphite, and 10 wt % of polymer binder (polyvinylidene fluoride, PVDF) (Arkema Canada Inc., Bécancour, Canada), which were then pasted on aluminum foil, followed by drying under a vacuum at 110 °C for 12 h. The active material loading in each electrode disc (about 12 mm in diameter) was typically 3.0–3.5 mg. The lithium disc served as both counter electrode and reference electrode; 1 M LiPF_6_ in a mixture of ethylene carbonate (EC) and dimethyl carbonate (DMC) (1:1, *v*/*v*) was used as an electrolyte and the separator was Celgard 2400. The galvanostatic charge/discharge measurements were performed using a Neware battery tester (BTS-4000) (Neware, Shenzhen, China) at different current densities with a cut-off voltage window of 2.2–4.2 V vs. Li/Li^+^. All electrochemical measurements were performed at room temperature.

## 3. Results and Discussion

### 3.1. Physical Characterizations

The microstructures of the LFP/graphene composites were investigated by scanning electron microscopy (SEM) and transmission electron microscopy (TEM) at different magnifications, and the results are presented in Figure 1b–g. In Figure 1b–g, it reveals a quasi-spherical morphology with a size of around 200–800 nm in diameter for all the pristine LFP samples. Meanwhile, a random aggregation phenomenon of LFP primary nanoparticles can be observed in Figure 1b. For the LFP/graphene samples, it can be clearly seen that the LFP particles are in contact with graphene to constitute a conducting network structure that can promote the electronic and ionic transport to improve the rate and cyclic performance of olivine-type LFP [23]. However, different size and morphology of graphenes with LFP show different constructions. In Figure 1c, the home-made graphene nanosheets are embedded into the LFP particles, which will contribute to high-performance batteries. From Figure 1d,e, it can be clearly seen that the size of G V20 is larger than that of G V4, so that the LFP particles can disperse on/in G V20 nanosheets uniformly with less LFP particle aggregation.

**Figure 2:**
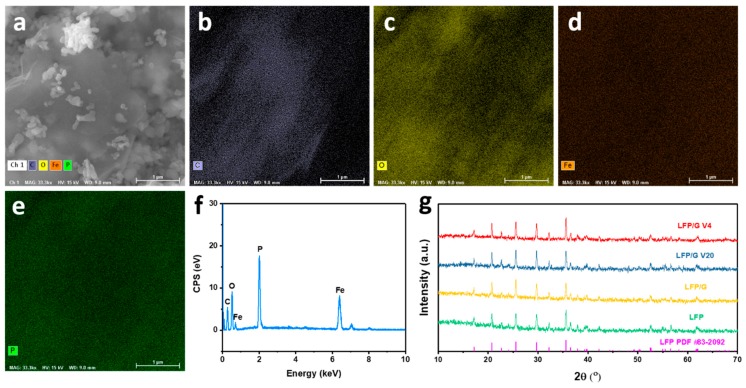
(**a**–**e**) EDX mapping images and (**f**) EDX spectrum of LFP/graphene. (**g**) XRD patterns of LFP, LFP/G, LFP/G V20, and LFP/G V4 samples.

A high-resolution TEM image (HRTEM, in Figure 1f) taken on an individual nanoparticle of quasi-spherical LFP displays clear crystal planes with d-spacings of 0.42 and 0.3 nm, corresponding to the (101) and (211) planes of orthorhombic LFP [31]. In addition, from Figure 1f,g, an amorphous carbon layer with a thickness of 3–4 nm covering the surface of the LFP particles can be clearly observed. That aside, the graphene can also be clearly seen for LFP deposition. The inset in Figure 1g is the indexed fast Fourier transform (FFT) pattern of Figure 1f, which also indicates the single crystal characteristics of the LFP nanoparticles. The energy dispersive X-ray spectroscopy (EDX) mapping images and EDX pattern of LFP/graphene in Figure 2a–f further confirms the successful incorporation of LFP and graphene.

The phase composition and structures of the prepared samples were identified by X-ray powder diffraction (XRD). Figure 2g compares the XRD patterns of LFP, LFP/G, LFP/G V20, and LFP/G V4 composites. For all samples, all Bragg peaks of samples can be well indexed as an olivine phase with an ordered orthorhombic structure belonging to the Pnma space group (PDF #83-2092), indicating a high crystallinity of the synthesized samples. In addition, no evidence of diffraction peaks for graphene and/or carbon appears in the diffraction pattern and its presence does not influence the structure of LFP, which could be ascribed to the low contents of graphene and/or carbon in the composites.

### 3.2. Electrochemical Performance

**Figure 3:**
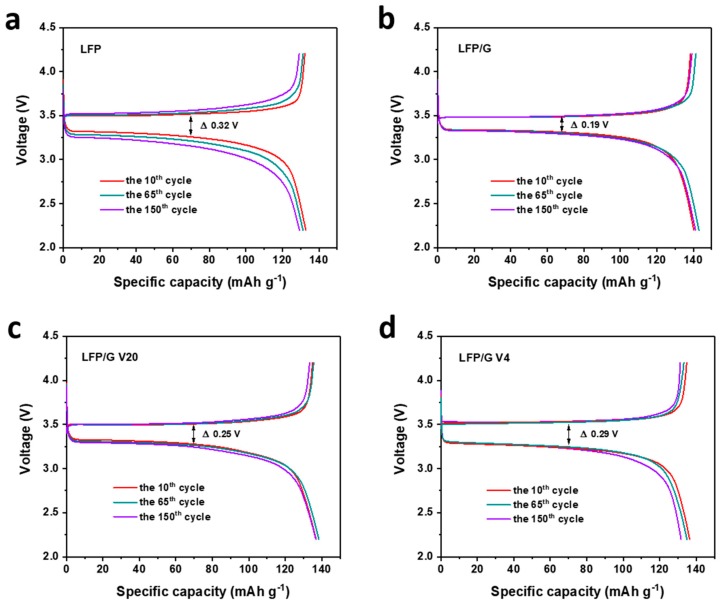
The charge/discharge profiles of (**a**) LFP, (**b**) LFP/G, (**c**) LFP/G V20, and (**d**) LFP/G V4 samples at 1 C.

In order to evaluate the electrochemical performance of the prepared samples, Figure 3 shows the charge/discharge profiles of all the samples at 1 C (1 C = 170 mA g^−1^). Naturally, they exhibit negligible capacity loss after 150 cycles, indicating excellent cycling stability. That aside, the potential differences between the voltage plateaus of charge and discharge profiles of all the samples can be described as: LFP/G ˂ LFP/G V20 ˂ LFP/G V4 ˂ LFP. The larger gap between the charge and discharge plateaus is probably caused by the inferior conductivity of the electrode material. Compared with bare LFP electrodes, the polarizations of the LFP/graphene electrodes upon cycling are smaller, benefiting from the superior electrical conductivity by the addition of graphene to LFP.

**Figure 4:**
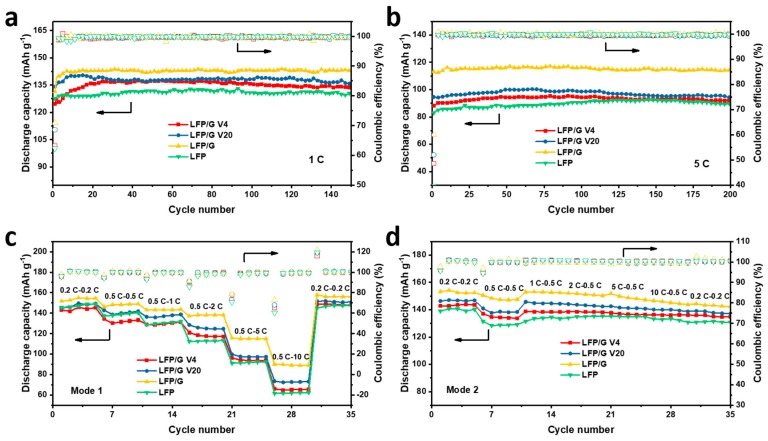
The cycling performance of LFP, LFP/G, LFP/G V20, and LFP/G V4 samples at (**a**) 1 C and (**b**) 5 C. The rate performance of LFP, LFP/G, LFP/G V20, and LFP/G V4 samples under various rates. (**c**) Mode 1: Discharge rate ≥ 0.5 C, charge rate: 0.5 C and (**d**) Mode 2: Charge rate ≥ 0.5 C, discharge rate: 0.5 C.

Figure 4a compares the cycling performance of all the samples at 1 C. The capacities stabilize at certain values, indicating good stability. Due to the addition of graphene, the specific capacities of LFP/G, LFP/G V20, and LFP/G V4 electrodes after 150 cycles are approximately 142, 139 and 136 mAh g^−1^, respectively, which are all higher than 130 mAh g^−1^ of pristine LFP and represent an increase of up to about 4.6–9.2%. The initial Coulombic efficiencies (CE) of LFP/G, LFP/G V20, and LFP/G V4 are 70.9%, 68.6%, and 63.2%, respectively, which are higher than 62.1% of LFP. After the first cycles, the CEs are stable and approach 100% upon cycling. The cycling performance of samples at 5 C is also shown in Figure 4b, and their corresponding initial CEs are 60.2%, 52.1%, 48.5%, and 39.6% for LFP/G, LFP/G V20, LFP/G V4, and LFP, respectively. Benefitting from the high electrical conductivity and high surface area of graphene, the specific capacities of LFP/G, LFP/G V20, and LFP/G V4 electrodes are higher than that of pristine LFP, indicating the importance and significance of adding graphene to LFP. Among these samples, the LFP/G shows the highest capacity of all the samples. 

**Figure 5:**
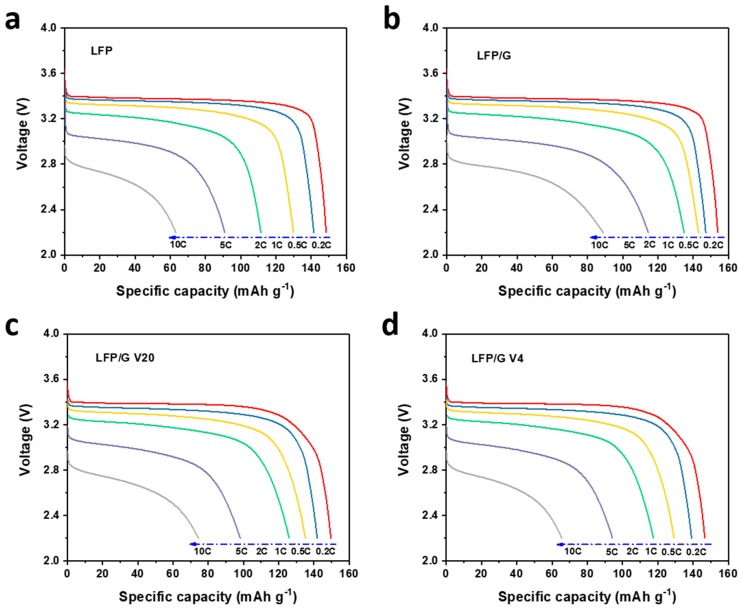
The discharge profiles of (**a**) LFP, (**b**) LFP/G, (**c**) LFP/G V20, and (**d**) LFP/G V4 samples at various rates (Mode 1: discharge rate ≥ 0.5 C, charge rate: 0.5 C).

The rate performances were tested with different modes, namely, Mode 1 and Mode 2. Mode 1 means that the cells are charging at 0.5 C when the discharging rate increases from 0.5 C to 10 C, i.e., slow charge; while Mode 2 means that the cells are discharging at 0.5 C when the charging rate increases from 0.5 C to 10 C, i.e., fast charge, which is also an important index of battery performance for practical applications. Figure 4c shows the rate capability of samples at various discharge rates of 0.2 C, 0.5 C, 1 C, 2 C, 5 C, and 10 C under Mode 1. These curves present typical gradients, and after quite a high current rate of 10 C, they still can return to their original capacities at a rate of 0.2 C. The CEs of the first cycle at each rate are different for electrodes, i.e., the LFP with graphene additives displays higher CEs than LFP. In addition, the CEs of the first cycle from 0.2 to 10 C decrease with the increase of cycling rates. The two-dimensional graphene exhibits remarkably high electronic mobility, such that charge carriers in this one-atom-thick material can travel ballistically over submicron distances [22,23,32]. Hence, the LFP/graphene electrodes exhibit a higher rate capacity than the LFP electrode, indicating graphene is an excellent agent/additive to improve the electrical conductivity of an electrode. The corresponding discharge profiles of samples at various rates are shown in Figure 5. Apparently, with the increase of the discharge current rate, the LFP samples with graphene exhibit less capacity decrease, especially the LFP/G, which is ascribed to the improved electrical conductivity by graphene. Overall, the developed LFP/graphene composites display excellent performance (good stability and high capacity) compared with the LFP, indicating that the incorporated graphene could dramatically improve the surface electrical conductivity of LFP nanoparticles and decrease the polarization resistance of the cathode. That aside, different sizes and morphology of graphene could possibly affect the electrochemical performance of electrodes.

With the fast growing demands to shorten the charging time of practical applications in our daily life, such as portable electronics and electric vehicles, achieving fast charging in energy storage systems, especially the widely used LIBs, has been strongly considered as the most important criterion for practical applications [33,34]. As for the function of fast charge, performance at various charge rates is a very important index of batteries. Therefore, the effect of charge rate on the performance of samples was also tested at various discharge rates of 0.2 C, 0.5 C, 1 C, 2 C, 5 C, and 10 C under Mode 2. Unlike Mode 1, when the charge rate is 1 C or even bigger, these curves don’t display typical gradients but gradual capacity fading with a very small fading rate, as the increase of rates shown in Figure 4d. Furthermore, unlike Mode 1, the CEs of the first cycle for electrodes after 0.5 C of charging show no obvious decrease with the increase of cycling rates, suggesting potential fast charging for practical applications.

**Figure 6:**
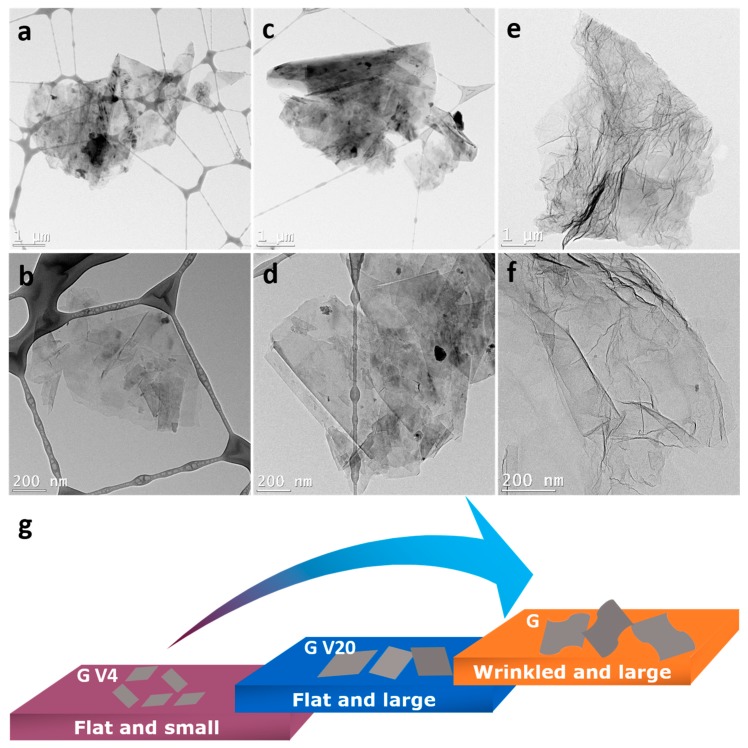
TEM images of (**a**,**b**) G V4, (**c**,**d**) G V20, (**e**,**f**) home-made G. (**g**) Illustration of features of G V4, G V20, and home-made G.

Overall, from the above-demonstrated electrochemical performance, the LFP with different sizes and morphology of graphene displays a varied performance in LIBs, which shows that the graphene shape also plays an important role in capacity output. To figure out the effect of different types of graphene on the electrochemical performance of LFP, TEM images of these three graphene were obtained in Figure 6a–f. From the figure, it is clear that the company graphene (G V4 and G V20) are flat, while the home-made G is wrinkled. Regardless, the size of graphene is ordered: G V4 < G V20 < home-made G, as illustrated in Figure 6g. Therefore, considering the features of the graphene used in this work, the reasons for the home-made G and the G V20 with better performance can be: (i) larger and curved home-made G could be uniformly dispersed between LFP particles to construct the conductive network; (ii) for the G V20 with relatively larger pieces of graphene, more conductive sites are formed in the electrode, which means more links between the spots of LFP and surface of graphene to enhance the conductivity, leading to better battery performance than G V4; (iii) for the G V4, the relatively smaller size (compared with G V20) makes it scatter in isolation among the LFP particles, resulting in inferior conductivity because of the non-continuous conductive network. 

**Figure 7:**
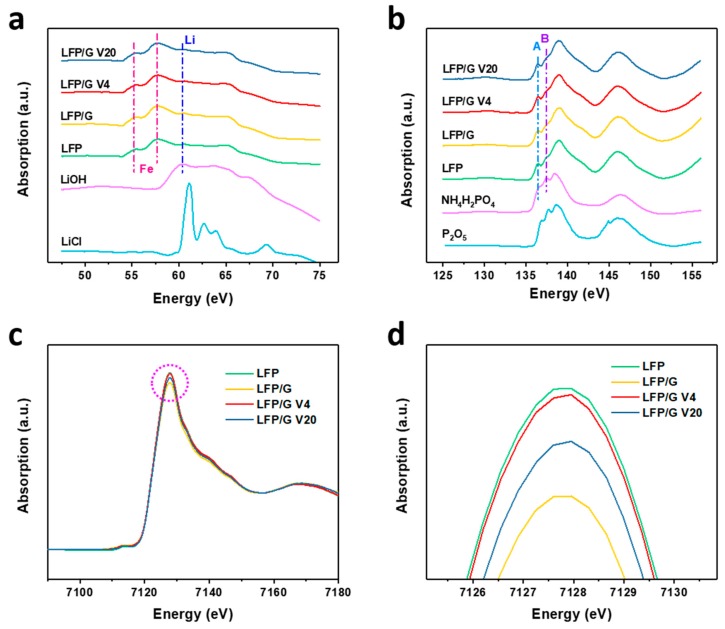
Total electron yield (TEY) of (**a**) Li K-edge and (**b**) P L-edge of the compounds. (**c**) Fe K-edge X-ray absorption near edge structure (XANES) spectra of the samples, and (**d**) the enlarged view of the circle in (**c**).

Furthermore, XANES measurement was conducted to compare the difference between these samples. Figure 7a shows the Li K-edge XANES spectra for lithium-containing compounds, including LFP samples in this work, LiCl, and LiOH. A sharp edge jump after 60 eV can be clearly observed for LiCl and LiOH [35]. Since the photon energy of the M-edges of Fe is very close to the Li K-edge, the features of Li in LFP and LFP/G samples are not as prominent as in reference samples. The electronic transitions can be allocated to Fe M_3,2_-edges (below 60 eV) and the Li K-edge (above 60 eV), respectively. Figure 7b shows the P L-edge XANES spectra for phosphorous-containing compounds, including LFP samples in this work, NH_4_H_2_PO_4_, and P_2_O_5_. The features of a P L_2,3_-edge spectrum are generally described by a doublet resonance. These two peaks are due to transitions from spin–orbit split 2p electrons (the 2p_3/2_ and 2p_1/2_ levels) into the first unoccupied 3s, like an antibonding state. The intensity of peak B and peak A (I_B_/I_A_) in these lithium phosphates arises from the distortion of the phosphate tetrahedron [36]. The difference between LFP and NH_4_H_2_PO_4_ indicates that there is more charge redistribution between the phosphate ion and Fe in LFP. By carefully comparing, we found that the I_B_/I_A_ of LFP was slightly lower than the other graphene-modified LFP, which demonstrates that the graphene slightly raised the degree of disorder of the phosphate tetrahedron in LFP. The Fe K-edge XANES spectra (Figure 7c) consist of two main edge jumps, the pre-edge and the main edge regions. The pre-edge peak is centered at the lower energy side of the sharply rising absorption edge (white line), corresponding to the 1s-to-3d electronic transition of Fe. All of the LFP composites exhibit a distinct increased white line located at about 7126 eV, and Fe valence in all LFP samples show a typical-II state [37]. From the enlarged view of the peak area in Figure 7d, we can find that the peak area follows the order: LFP > LFP/G V4 > LFP/G V20 > LFP/G, which means that the charge transfer among these samples follows the same order, being consistent with the battery performance. This result shows that the less the charge transfer of Fe in the sample, the better the performance of the sample.

**Figure 8:**
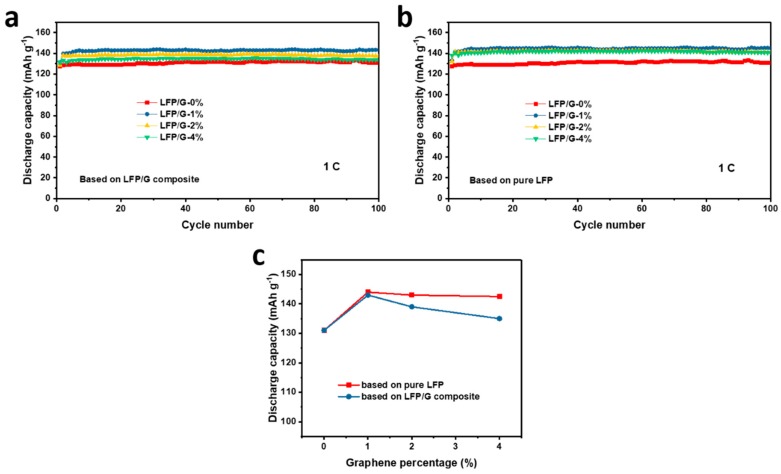
The cycling performance of LFP with different graphene contents ranging from 0% to 4%: (**a**) Capacity calculated based on the LFP/G composite and (**b**) capacity calculated based on the pristine LFP. (**c**) The plot of graphene percentage and discharge capacity.

From the point of view of high energy density and unwanted voltage polarization, the carbon additives to LFP should be as low as possible [23]. In this work, in order to investigate the effect of graphene content on electrochemical performance of LFP/graphene composites, electrodes with various contents of home-made graphene (ranging from 0% to 4%) were prepared and tested. The cycling performance of LFP with different graphene contents is shown in Figure 8a. Among these samples, the LFP/G-1% shows the best cycling performance, which indicates the optimized graphene content in LFP composites. The above results were calculated based on the total weight of LFP/G composites. For the capacity calculated based on pure LFP, the corresponding results are shown in Figure 8b. Unlike the results in Figure 8a, the discharge capacity of LFP/G with 1%, 2%, and 4% almost stabilizes at the same value during cycling, which demonstrates that increasing the graphene content would not improve the capacity of LFP significantly. Figure 8c shows the corresponding tendency of the effect of graphene content on the discharge capacity of LFP electrodes. With low graphene content, increasing the amount of graphene can improve the discharge capacity, while larger content (> 1%) will not influence the electrochemical performance of LFP significantly, though it lowers the capacity of the complete electrode. Moreover, with regards to the economic and electrochemical performance, 1% of graphene content is optimized, no matter the capacity calculated based on the LFP/graphene composite or pure LFP in this work. 

## 4. Conclusions

The electrochemical performance of the LFP sample with the addition of graphene (i.e., home-made G, as well as NanoXplore company graphene (heXo-G V4 and heXo-G V20)) were investigated, using button-type coin cells. Except for the home-made G, all of the LFP, G V4, and G V20 were sourced from industrial manufacturers, which is favorable for commercialization. Comparing the physical characterizations and electrochemical performance, we observed that with the additional graphene, the LFP/graphene exhibited much more stable cyclic performance, higher capacity, and better rate performance than their counterparts without graphene, suggesting the additional graphene could improve the electrochemical performance of LFP for batteries. Meanwhile, the addition of graphene did not affect the olivine structure of LFP. Therefore, LFP/graphene composites hold potential interest as cathode materials in high-performance lithium-ion batteries for EVs and HEVs. Electrochemical performance of commercial grade graphene also showed promise in next-generation low-cost LFP/graphene cathodes for industrial use. Moreover, by comparing the electrochemical performance of LFP with different types of graphene, it was found that the intrinsic features of graphene could be able to significantly affect performance, and that the wrinkled and large graphene nanosheet is superior to the flat and small graphene. Capacity calculated based on different methods has varying results. In this work, we also demonstrated that from the perspective of economic and electrochemical performance, 1% of graphene content is optimized no matter the capacity calculated, based on the LFP/graphene composite or pure LFP.
